# A New *T. gondii* Mouse Model of Gene-Environment Interaction Relevant to Psychiatric Disease

**DOI:** 10.1155/2018/7590958

**Published:** 2018-12-02

**Authors:** Geetha Kannan, Emese Prandovszky, Emily Severance, Robert H. Yolken, Mikhail V. Pletnikov

**Affiliations:** ^1^Department of Psychiatry and Behavioral Sciences, Johns Hopkins University School of Medicine, Baltimore, MD 21287, USA; ^2^Department of Microbiology and Immunology, University of Michigan Medical School, Ann Arbor, MI 48109, USA; ^3^Stanley Neurovirology Laboratory, Department of Pediatrics, Johns Hopkins University School of Medicine, Baltimore, MD 21287, USA; ^4^The Solomon H. Snyder Department of Neuroscience, Johns Hopkins University School of Medicine, Baltimore, MD 21205, USA

## Abstract

Infection with the protozoan parasite, *Toxoplasma gondii* (*T. gondii*), was linked to several psychiatric disorders. The exact mechanisms of a hypothesized contribution of *T. gondii* infection are poorly understood, and it appears that only a subset of seropositive individuals go on to develop a mental illness, suggesting genetic vulnerability. In order to stimulate mechanistic studies of how exposure to *T. gondii* could interact with genetic predisposition to psychiatric disorders, we have generated and characterized a mouse model of chronic *T. gondii* infection in BALB/c mice with inducible forebrain neuronal expression of a C-terminus truncated dominant-negative form of disrupted-in-schizophrenia 1 (DN-DISC1). In this gene-environment interaction (GxE) model, exposing control and DN-DISC1 male and female mice to *T. gondii* produced sex-dependent abnormalities in locomotor activity and prepulse inhibition of the acoustic startle. No genotype- or sex-dependent effects were found on levels of anti-Toxoplasma IgG antibodies or anti-NMDAR or C1q antibodies. Our work demonstrates that a psychiatric genetic risk factor, DN-DISC1, modulates the neurobehavioral effects of chronic *T. gondii* infection in a sex-dependent manner. The present *T. gondii* model of GxE provides a valuable experimental system for future mechanistic studies and evaluation of new treatments.

## 1. Introduction

There has been a growing interest in the role of infectious agents in the development of psychiatric disorders. Epidemiological and immunological studies have identified microbial factors that may contribute to major psychiatric disorders, including schizophrenia and bipolar disorder [1, 2]. However, the mechanisms whereby microbes could affect the brain and behavior development remain incompletely understood. The consensus in the field is that both direct and indirect mechanisms could be at play to explain behavioral pathological changes observed in the infected host [3].

In order to advance our understanding of the mechanisms whereby microbes contribute to the pathophysiology of mental disease, most animal models have focused on indirect mechanisms that include the innate and/or adaptive immune responses to infectious agents [4, 5]. Assuming that most viral and/or bacterial pathogens activate similar immune signaling pathways, these studies have used synthetic immune stimulators to mimic viral (poly I : C) or bacterial (LPS) infection [6–8]. There have been generated very few animal models utilizing live pathogens. The major impediments for progress in generating appropriate animal models have been species-specific differences, increased mortality rate in rodents or the inability of a human virus or bacterium to replicate in the animal host without manipulating the microbe's genome or the host's immune system (e.g., HSV-1) [9]. In this context, *Toxoplasma gondii* (*T. gondii*), a protozoan parasite found world-wide, offers a unique opportunity for generating infectious animal models of human psychopathology as this parasite utilizes similar, if not same, mechanisms for its replication and dissemination in animals and humans [10].

Although exposure to *T. gondii* exposure (i.e., seropositivity) was associated with major psychiatric disorders [2, 10], including schizophrenia and bipolar disorder, we know very little why only a subset of seropositive subjects are diagnosed with a psychiatric disease. While genetic variability of the parasite's genome and host's age can modify behavioral pathology of the infected host [11–13], the role of the host's genetic susceptibility remains practically unstudied. The only animal study has shown that deletion of a gene involved in dopamine synaptic neurotransmission, *Nurr1*, could alter the behavioral changes produced by chronic *T. gondii* infection in mice [14]. No mouse model carrying a human psychiatric risk variant has been generated to evaluate possible gene-environment interaction (GxE) relevant to psychiatric disorders associated with exposure to *T. gondii*. In order to model GxE in mice, we have developed a new mouse model of inducible expression of C-terminus truncated form of disrupted-in-schizophrenia-1 (DISC1).

*DISC1* is a gene disrupted by the balanced (1 : 11) (q42.1; q14.3) translocation, segregating in the Scottish family with several major psychiatric disorders, including schizophrenia, depression, and bipolar disorder [15–17]. Although the DISC1 locus has not been reported in the latest genome-wide association studies [18], rare mutations of large effects contribute to mental disorders [19] and are critical for uncovering the molecular pathobiology of psychiatric disease [20, 21]. It is in this context that we consider DISC1 as a major neurodevelopmental risk factor. The Scottish translocation may result in *DISC1* haplo-insufficiency or production of mutant DISC1 protein that could act in a dominant-negative manner [6, 16]. Both outcomes seem to lead to a similar disturbance in DISC1-interacting protein complexes and a loss of DISC1 function [6]. Thus, we use a C-terminus-truncated form of full-length protein as a dominant-negative molecular tool (DN-DISC1) to alter (i.e., decrease) expression of endogenous full-length DISC1 in order to elucidate the role of DISC1 in our *T. gondii* mouse model of GxE.

We found that chronic *T. gondii* infection of DN-DISC1 mice led to an abnormal decrease in general locomotor activity and impaired prepulse inhibition of acoustic startle in a sex-dependent manner. The present study describes a new mouse model of GxE to better our understanding of how exposure to *T. gondii* could contribute to mental disease in human.

## 2. Methods and Materials

### 2.1. Animals

Male and female BALB/c mice (the Jackson Laboratory, Bar Harbor, ME) were used in this study. Mice were housed 2–4 per cage (initially 5 per cage) in the JHU animal facility with 14.5/9.5 hours of light/dark cycle and free access to food and water. Animal protocols were reviewed and approved by the Animal Care and Use Committee of Johns Hopkins University (JHU). All experiments conformed to the US National Institutes of Health *Guide for the Care and Use of Laboratory Animals*, and all efforts were made to minimize the number of mice used and their suffering.

### 2.2. DISC1 Mouse Model

We used transgenic (Tg) mice that express C-terminus truncated disrupted-in-schizophrenia-1 (DISC1), a putative product of the Scottish balance translocation [22–24]. Previous studies have shown that truncated human DISC1 acts as a dominant negative factor (DN-DISC1) on the background of full complement of endogenous mouse DISC1 [25]. To express DN-DISC1, we utilized the inducible Tet-Off model system as previously described [26]. Briefly, in Tg regulatory line, cell type-specific expression of the tetracycline transactivator (tTA) is driven by the CAMKII promoter to express tTA in forebrain pyramidal neurons. When tTA binds in pyramidal neurons to TetO sequences present on Tg tetracycline response element (TRE) responder line that carries downstream of the minimal CMV promoter the cDNA sequence of DN-DISC1, it leads to predominant expression of DN-DISC1 in forebrain pyramidal neurons [26].

This mouse model was originally generated on the C57BL/6 background [26]. In order to use this double Tg mouse model in combination with Toxoplasma infection, we transferred both transgenes (i.e., tTA and DN-DISC1) to the BALB/c background. In contrast to C57BL/6 mice, there is a lower level of mortality due to Toxoplasma infection in BALB/c mice, allowing the use of Tg mice infected with *T. gondii* in neurobiological and behavioral studies [27, 28].

Breeding heterozygous tTA-Tg mice with heterozygous TRE-DN-DISC1 mice produces ∼25% of mice that express DN-DISC1 (double Tg mice) and ∼25% of each of all other products of this breeding protocol (tTA^−^/DN-DISC1^−^; tTA^−^/DN-DISC1^+^; tTA^+^/DN-DISC1^−^). Based on our prior studies [26], we used double Tg mice as our experimental group (DN-DISC1 mice) and single Tg mice, tTA^−^/DN-DISC1^+^, as our control group (control mice). Tail tissue samples were used for genotyping as previously described [26]. Developing mice were housed with their dams until postnatal days (P) 21–23, with food and water provided ad libitum.

### 2.3. The Overall Experimental Design

The DN-DISC1 mouse model on the BALB/c background had never been evaluated before. Thus, we initially assessed a postnatal time course of expression of DN-DISC1 in the transgenic mice. A separate cohort of mice was genotyped and tested in a series of behavioral tests to identify possible baseline differences between control and DN-DISC1 male and female mice. Behavior testing began when mice were 7–15 weeks of age. After initial behavioral testing, the same mice were infected with *T. gondii* to determine the effects of chronic *T. gondii* infection on mouse behavior. Mice were infected with *T. gondii*, as described below, at 13–19 weeks of age. Mice were then retested in behavioral paradigms at 21–27 weeks of age (8 weeks after infection). Upon completion of behavioral testing, mice were sacrificed, and their serum samples were assayed for titers of antibodies to *T. gondii*, the *N*-methyl-d-aspartate receptor (NMDAR) GluN2 subunit, and the first subcomponent of the C1 complex (C1q).

### 2.4. Behavioral Testing

For both pre- and postinfection behavioral testing, novelty-induced activity was assessed prior to evaluation of sensorimotor gating.

### 2.5. Novelty-Induced Activity

Novelty-induced activity in the open field was assessed over a 30-minute period using activity chambers with infrared beams (San Diego Instruments Inc., San Diego, CA, USA). Horizontal and vertical activities were automatically recorded.

### 2.6. Sensorimotor Gating

Prepulse inhibition of the acoustic startle (PPI) was used to assess sensorimotor gating. Mice were acclimatized in startle chambers (San Diego Instruments, Inc., San Diego, CA) to a 76 dB background noise (continuous throughout the session) for 5 min, followed by the presentation of ten 40 ms 120 dB white noise stimuli at 20 s interstimulus intervals (habituation). After habituation, mice were left in the enclosure for 5 min without presentations of any startle stimuli. Immediately after this, the prepulse inhibition (PPI) session was begun, consisting of five presentations of each trial type in a pseudorandom order. The types of trials include pulse-alone (120 dB, 100 msec, broadband burst), omission of stimuli, and five pre-pulse-pulse combinations consisting of a 20 msec prepulse broadband burst at either 77, 78, 80, 84, or 88 dB, presented 80 msec before the pulse. Presentation intervals varied from 10 to 19 seconds. Mean PPI percentage was calculated by averaging all PPI values for presentations of all prepulses for each experimental group. These values were used for graphing and statistical analysis. Startle reactivity was evaluated by comparing absolute values of the 120 dB startle amplitude.

### 2.7. Toxoplasma Infection

Prugniaud (PRU) tachyzoites (≤passage 6 *in vitro*) were maintained by passage in human foreskin fibroblast (HFF) monolayers and purified as previously described [29]. All mice were infected with 400 tachyzoites in 200 *μ*L of 1x PBS (2 parasites/*μ*L) intraperitoneally (IP) at 13–19 weeks of age using 26-gauge needles. Each mouse was gently picked up by an experimenter, and a single IP injection was quickly performed to minimize any possible stressful effects of the injection.

### 2.8. Western Blotting Analysis of DN-DISC1 Expression

Mouse forebrain samples from control (tTA^−^/DN-DISC1^+^) or DN-DISC1 (double Tg mice) mice were collected at embryonic day 17.5 (E17.5), postnatal day 1 (P1), and P77 to measure expression of DN-DISC1 by western blotting. Isolated tissue samples were homogenized with a pestle in lysis buffer composed of RIPA buffer (SIGMA) and 1x protease inhibitor (Sigma). Protein (25 *μ*g/well) was separated on NuPage 4–12% Bis-Tris, 1.0 mm gels (Invitrogen) and transferred onto nitrocellulose 0.45 *μ*m (BioRad) membranes. The membrane was probed with anti-*myc* (a tag fused with DN-DISC1, 1 : 2000, Roche), and *β*-tubulin (1 : 10,000; Sigma) was used as the loading control. As the goal of this experiment was to determine the presence of DN-DISC1 in double transgenic mice and lack of expression in single transgenic mice, no quantification of expression was done.

### 2.9. Immunoassays

Using enzyme-linked immunosorbent assays, serum IgG antibodies were measured for *T. gondii*, the *N*-methyl-d-aspartate receptor (NMDAR) GluN2 subunit, and the first subcomponent of the C1 complex (C1q) as previously described [30, 31]. Each 96-well plate tested contained kit standards as well as study sample replicates for use as internal controls of reproducibility.

### 2.10. Statistical Analysis

Novelty-induced locomotor activity and PPI were analyzed with two-way ANOVA, with infection and genotype as independent variables and locomotor activity or PPI value as dependent variables, respectively. Serology data were analyzed with one-way ANOVA or the Student two-tailed *t*-test. A value of *P* ≤ 0.05 was considered significant. All data are presented as means ± standard error of means (SEM).

## 3. Results

### 3.1. Expression of DN-DISC1 in BALB/c Mice

**Figure 1:**
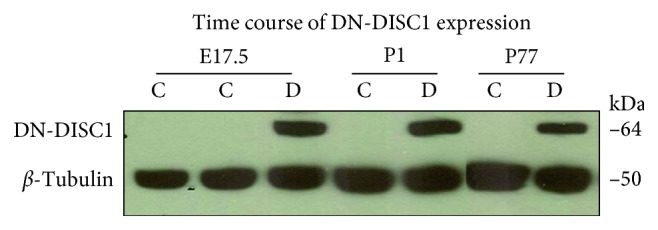
Time course of DN-DISC1 expression in the mouse brain across neurodevelopment. The mouse forebrain was isolated at embryonic day 17.5 (E17.5), postnatal day 1 (P1), and postnatal day 77 (P77) in control (C) and DN-DISC1 (D) mice. Tissue samples were prepared for western blotting to evaluate expression of DN-DISC1 (64 kDa) using anti-*myc* antibody (a tag for DN-DISC1) and tubulin as a loading control. No quantitative assessment of age-dependent expression was made. Each sample was run in triplicate.

Our data show that we successfully transferred both transgenes to the BALB/c background (Figure 1). Consistent with our prior studies [26], we found expression of DN-DISC1 starting at late gestation stage and until adulthood.

### 3.2. Effects of GxE on Locomotor Activity

**Figure 2:**
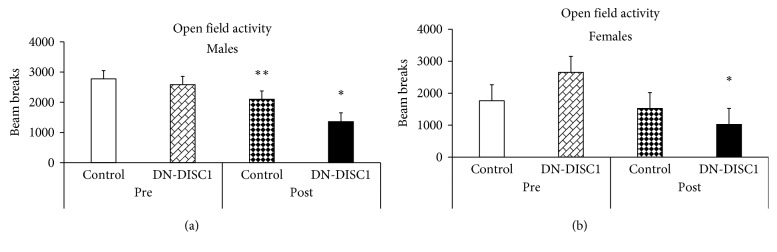
Effects of GxE on locomotor activity. Locomotor activity was assayed in open field test for 30 minutes in control and DN-DISC1 male (a) and female (b) mice; *n* = 9-10 per group for male mice; *n* = 5 per group for female mice. Two-way ANOVA of the total activity data showed a significant effect of infection for male mice, *F*(1,37) = 11.3, *p*=0.002; and for female mice, *F*(1,19) = 7.2, *p*=0.017. No effects of the genotype or the genotype by infection interaction were found for either sex. ^*∗*^*p* < 0.05 vs. uninfected mice of the same genotype; ^*∗∗*^*p* < 0.05 vs. infected DN-DISC1 male mice.

Preinfection behavioral tests were performed on 7–15-week-old control and DN-DISC1 male and female BALB/c mice. We found no significant effects of DN-DISC1 on total horizontal locomotor activity in female or male mice (Figure 2). The same control and DN-DISC1 male and female mice were exposed to *T. gondii* at 13–19 weeks of age and retested in the same 8 weeks after infection, a time when *T. gondii* has encysted in the brain. There was a significant decrease in locomotor activity in infected male and female DN-DISC1 mice compared with respective uninfected control groups (Figure 2). When compared to infected control male mice, infected DN-DISC1 male mice produced a significant reduction in locomotion (Figure 2). Such a phenomenon was not noted in infected DN-DISC1 female mice.

### 3.3. Effects of GxE on PPI

**Figure 3:**
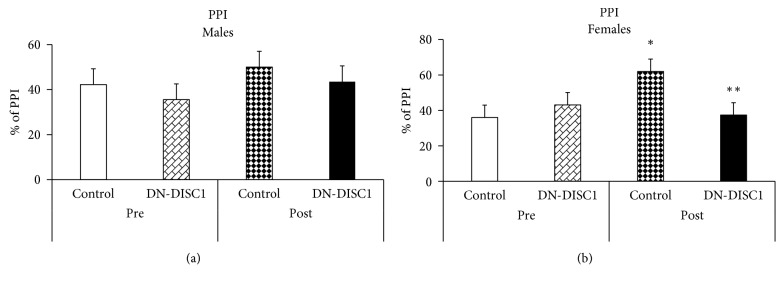
Effects of GxE on PPI. PPI of the acoustic startle was assayed in control and DN-DISC1 male (a) and female (b) mice; *n* = 9-10 per group for male mice; *n* = 5 per group for female mice. Two-way ANOVA of PPI data for male mice showed no effects. Two-way ANOVA for female mice showed a significant genotype by infection interaction, *F*(1,19) = 10.1, *p*=0.038; ^*∗*^*p* < 0.05 vs. uninfected control female mice; ^*∗∗*^*p* < 0.05 vs. infected control female mice.

Similar to the open field data, no significant effects of DN-DISC1 were noted on the acoustic startle or prepulse inhibition (PPI) of the acoustic startle in male or female mice (Figure 3). No effects of infection on PPI were found in male mice of either genotype. Compared to uninfected control female mice, *T. gondii* increased PPI in infected control female mice. Compared to uninfected control female mice, infected DN-DISC1 female mice exhibited significantly decreased PPI (Figure 3). No infection-dependent alterations in startle response were noted, suggesting that altered PPI in female mice was unlikely related to changes in startle responsiveness (data not shown).

### 3.4. Serological Findings

Our serological studies did not reveal any effects of DN-DISC1 on anti-Toxo IgG levels (Table 1). No effects of DN-DISC1 or *T. gondii* on titers of anti-GluN2 or C1q antibodies were found either (Table 2). Statistical analyses did not show any significant differences in all those measures.

## 4. Discussion

The present study is the first one to evaluate interaction between chronic *T. gondii* infection and a rare but highly penetrant mutation associated with major psychiatric disorders. Our findings indicate that neurobehavioral consequences of chronic *T. gondii* infection in mice could be modulated by genetic risk factors associated with psychiatric disease.

The sex-dependent abnormalities we observed in *T. gondii*-infected DN-DISC1 mice are consistent with those reported for other GxE models. Eells and associates have found that *T. gondii* infection of *Nurr1* heterozygous male mice exacerbated elevated open field activity that correlated with antibody titers of infected mice [14]. In another study, *Neuregulin 1* knockout (KO) female but not male mice showed enhanced cued-fear extinction following stress exposure [32, 33]. While no sex-related differences between WT and *Comt* KO mice were observed in the basic acquisition of the five-choice serial reaction time task, mild stress adversely affected cognitive performance in COMT knockout males, but not in females and revealed other complex sex-dependent effects in this GxE mode [34]. Using DN-DISC1 model generated on the C57BL6, we have demonstrated that a lifelong exposure of DN-DISC1 mice to Pb^2+^ produced hyperactivity, exaggerated responses to the NMDAR antagonist, MK-801, mildly impaired prepulse inhibition of the acoustic startle, and enlarged lateral ventricles in female DN-DISC1 mice only [35]. Similarly, in response to combined neonatal poly I : C and peripubertal unpredictable stress, adult female rats showed more pronounced PPI deficits, while males showing greater alterations in social interaction [36]. As there were no mutation- or sex-dependent changes in titers of anti-IgG antibodies, it seems unlikely that sex-dependent effects of *T. gondii* in DN-DISC1 mice were related to differences in the peripheral immune response. Future research on G × E models should address the underlying mechanisms of sex-specific abnormalities [37, 38].

Sex-dependent effects of *T. gondii* infection have been also reported in a few human studies. Lindová et al. found gender effects on the personality traits [39]. Although the exact mechanisms underlying sex-dependent effects of *T. gondii* remain unclear, there is evidence that subjects with latent infection have altered concentration of testosterone. Flegr et al. found that Toxoplasma-exposed men had a higher concentration of testosterone while Toxoplasma-exposed women had a lower concentration of testosterone compared to gender-appropriate Toxoplasma-unexposed control subject [40].

Our prior studies have demonstrated that chronic *T. gondii* infection leads to increased titers of anti-NMDAR and anti-C1q antibodies [30] that were implicated in several neurological and psychiatric conditions in humans [41–43]. As we observed no GxE effects on these serological measures, one can suggest that these pathogenic antibodies might not be involved in the behavioral pathology exhibited by infected DN-DISC1 mice. Given the role of DISC1 in regulating major inflammatory singling in the cell (e.g., NF-kB) [44, 45], it is tempting to speculate that exaggerated response of DN-DISC1 mice to *T. gondii* could be at least in part explained by “primed” NF-kB signaling in DN-DISC1 mice. Future studies will test the above mechanistic hypotheses in detail.

We believe that the present work significantly advances research on GxE rodent models by utilizing live infection as a relevant environmental factor. We think this is a critical issue as using live human pathogens in animal models has been significantly impeded by species-specific pathogen-host interaction. For example, while HSV-1, CMV, or influenza virus was associated with psychiatric disorders and/or cognitive dysfunction [46, 47], recreating these viral infections in rodents has been proved to be difficult. For mice or rats to be infected and/or not to succumb to infection, either the host immune system or the microbe genome has to be manipulated to overcome the species barriers [48]. As *T. gondii* infection utilizes the similar, if not same, mechanisms for replication and dissemination in the majority of hosts, *T. gondii* rodent models, enable us to study the molecular pathogenesis of neurobehavioral abnormalities in rat or mice with reasonable confidence that similar pathophysiological processes are involved in humans.

Although in attempts to overcome the mentioned species-specific limitations, immune activation experimental systems have been introduced (e.g., poly I : C), this approach is not without limitations either because different microbial pathogens could stimulate different TRL receptors, with *T. gondii*, for example, activating TLR11 [49] that is not responsive to poly I : C or LSP [50]. In this context, our mouse model of chronic *T. gondii* infection provides another advantage for helping to sort out the direct and indirect mechanisms *T. gondii*-produced pathological and behavioral changes. In order to avoid use of artificial immune stimulators, our group has developed a valuable method of inactivating live parasites that retain the ability to evoke naturalistic IgG response without replicating in the brain [51].

The limitations of the present study include the small number of female mice and lack of a control group to evaluate possible stressful effects of IP injections in this model of GxE interaction. Future studies will address these limitations.

In conclusion, we show that a psychiatric genetic risk factor, DN-DISC1, modulates the neurobehavioral effects of chronic *T. gondii* infection in a sex-dependent manner. Our *T. gondii* model of GxE provides the valuable experimental system for future mechanistic studies and evaluation of new treatments of neurobehavioral abnormalities associated with this parasitic infection in humans.

## Figures and Tables

**Table 1:** *Toxoplasma gondii* IgG.

Group	Female	Male
Control	0.55 ± 0.12	0.64 ± 0.06
DN-DISC1	0.63 ± 0.08	0.75 ± 0.03

Data are mean IgG values ± SEM.

**Table 2:** Absence of group- or infection-related effects on anti-GluN or anti-Anti-C1q antibodies.

Group	GluN2		C1q	
	Male	Female	Male	Female
Control	0.28 ± 0.1	0.34 ± 0.06	0.18 ± 0.02	0.22 ± 0.02
DN-DISC1	0.41 ± 0.05	0.64 ± 0.29	0.22 ± 0.02	0.22 ± 0.06

Data are mean IgG values ± SEM.

## Data Availability

The data used to support the findings of this study are available from the corresponding author upon request.

## References

[1] Hertz-Picciotto I., Croen L. A., Hansen R., Jones C. R., van de Water J., Pessah I. N. (2006). The CHARGE study: an epidemiologic investigation of genetic and environmental factors contributing to autism. *Environmental Health Perspectives*.

[2] Yolken R., Torrey E. (2008). Are some cases of psychosis caused by microbial agents? A review of the evidence. *Molecular Psychiatry*.

[3] Ashdown H., Dumont Y., Ng M., Poole S., Boksa P., Luheshi G. (2006). The role of cytokines in mediating effects of prenatal infection on the fetus: implications for schizophrenia. *Molecular Psychiatry*.

[4] Dantzer R., O’Connor J. C., Freund G. G., Johnson R. W., Kelley K. W. (2008). From inflammation to sickness and depression: when the immune system subjugates the brain. *Nature Reviews Neuroscience*.

[5] Patterson P. H. (2009). Immune involvement in schizophrenia and autism: etiology, pathology and animal models. *Behavioural Brain Research*.

[6] Abazyan B., Nomura J., Kannan G. (2010). Prenatal interaction of mutant DISC1 and immune activation produces adult psychopathology. *Biological Psychiatry*.

[7] Meyer U., Nyffeler M., Yee B. K., Knuesel I., Feldon J. (2008). Adult brain and behavioral pathological markers of prenatal immune challenge during early/middle and late fetal development in mice. *Brain, Behavior, and Immunity*.

[8] O’connor J., Lawson M., Andre C. (2009). Lipopolysaccharide-induced depressive-like behavior is mediated by indoleamine 2, 3-dioxygenase activation in mice. *Molecular Psychiatry*.

[9] Stanberry L. (1992). *Pathogenesis of Herpes Simplex Virus Infection and Animal Models for Its Study: Herpes Simplex Virus*.

[10] Torrey E. F., Bartko J. J., Yolken R. H. (2012). *Toxoplasma gondii* and other risk factors for schizophrenia: an update. *Schizophrenia Bulletin*.

[11] Blader I. J., Saeij J. P. (2009). Communication between *Toxoplasma gondii* and its host: impact on parasite growth, development, immune evasion, and virulence. *APMIS*.

[12] Smith J. E., Rebuck N. (2000). *Toxoplasma gondii* strain variation and pathogenicity. *Microbial Foodborne Diseases: Mechanisms of Pathogenesis and Toxin Synthesis*.

[13] Xiao J., Jones-Brando L., Talbot C. C., Yolken R. H. (2011). Differential effects of three canonical Toxoplasma strains on gene expression in human neuroepithelial cells. *Infection and immunity*.

[14] Eells J. B., Varela-Stokes A., Guo-Ross S. X. (2015). Chronic *Toxoplasma gondii* in Nurr1-null heterozygous mice exacerbates elevated open field activity. *PLoS One*.

[15] Blackwood D. H. R., Fordyce A., Walker M. T., St. Clair D. M., Porteous D. J., Muir W. J. (2001). Schizophrenia and affective disorders—cosegregation with a translocation at chromosome 1q42 that directly disrupts brain-expressed genes: clinical and P300 findings in a family. *American Journal of Human Genetics*.

[16] Millar J. K., Christie S., Semple C. A. M., Porteous D. J. (2000). Chromosomal location and genomic structure of the human translin-associated factor X gene (TRAX; TSNAX) revealed by intergenic splicing to DISC1, a gene disrupted by a translocation segregating with schizophrenia. *Genomics*.

[17] St Clair D., Blackwood D., Muir W. (1990). Association within a family of a balanced autosomal translocation with major mental illness. *The Lancet*.

[18] Ripke S., Neale B. M., Corvin A. (2014). Biological insights from 108 schizophrenia-associated genetic loci. *Nature*.

[19] Farrell M., Werge T., Sklar P. (2015). Evaluating historical candidate genes for schizophrenia. *Molecular Psychiatry*.

[20] Geschwind D. H., Flint J. (2015). Genetics and genomics of psychiatric disease. *Science*.

[21] Sullivan P. F., Daly M. J., O’donovan M. (2012). Genetic architectures of psychiatric disorders: the emerging picture and its implications. *Nature Reviews Genetics*.

[22] Chubb J., Bradshaw N. J., Soares D., Porteous D., Millar J. (2008). The DISC locus in psychiatric illness. *Molecular Psychiatry*.

[23] Ishizuka K., Paek M., Kamiya A., Sawa A. (2006). A review of Disrupted-In-Schizophrenia-1 (DISC1): neurodevelopment, cognition, and mental conditions. *Biological Psychiatry*.

[24] Millar J., Christie S., Anderson S. (2001). Genomic structure and localisation within a linkage hotspot of Disrupted in Schizophrenia 1, a gene disrupted by a translocation segregating with schizophrenia. *Molecular Psychiatry*.

[25] Hikida T., Jaaro-Peled H., Seshadri S. (2007). Dominant-negative DISC1 transgenic mice display schizophrenia-associated phenotypes detected by measures translatable to humans. *Proceedings of the National Academy of Sciences*.

[26] Pletnikov M., Ayhan Y., Nikolskaia O. (2008). Inducible expression of mutant human DISC1 in mice is associated with brain and behavioral abnormalities reminiscent of schizophrenia. *Molecular Psychiatry*.

[27] Suzuki Y., Sher A., Yap G. (2000). IL-10 is required for prevention of necrosis in the small intestine and mortality in both genetically resistant BALB/c and susceptible C57BL/6 mice following peroral infection with *Toxoplasma gondii*. *Journal of Immunology*.

[28] Williams D., Grumet F., Remington J. (1978). Genetic control of murine resistance to *Toxoplasma gondii*. *Infection and Immunity*.

[29] Kannan G., Moldovan K., Xiao J. C., Yolken R. H., Jones-Brando L., Pletnikov M. V. (2010). *Toxoplasma gondii* strain-dependent effects on mouse behaviour. *Folia Parasitologica*.

[30] Kannan G., Crawford J. A., Yang C. (2016). Anti-NMDA receptor autoantibodies and associated neurobehavioral pathology in mice are dependent on age of first exposure to *Toxoplasma gondii*. *Neurobiology of Disease*.

[31] Severance E. G., Gressitt K. L., Halling M. (2012). Complement C1q formation of immune complexes with milk caseins and wheat glutens in schizophrenia. *Neurobiology of Disease*.

[32] Karl T. (2013). Neuregulin 1: a prime candidate for research into gene-environment interactions in schizophrenia? Insights from genetic rodent models. *Frontiers in Behavioral Neuroscience*.

[33] Taylor S. B., Taylor A. R., Koenig J. I. (2013). The interaction of disrupted Type II Neuregulin 1 and chronic adolescent stress on adult anxiety-and fear-related behaviors. *Neuroscience*.

[34] Papaleo F., Erickson L., Liu G., Chen J., Weinberger D. R. (2012). Effects of sex and COMT genotype on environmentally modulated cognitive control in mice. *Proceedings of the National Academy of Sciences*.

[35] Abazyan B., Dziedzic J., Hua K. (2013). Chronic exposure of mutant DISC1 mice to lead produces sex-dependent abnormalities consistent with schizophrenia and related mental disorders: a gene-environment interaction study. *Schizophrenia Bulletin*.

[36] Monte A. S., Mello B. S. F., Borella V. C. M. (2017). Two-hit model of schizophrenia induced by neonatal immune activation and peripubertal stress in rats: study of sex differences and brain oxidative alterations. *Behavioural Brain Research*.

[37] Hill R. A. (2016). Sex differences in animal models of schizophrenia shed light on the underlying pathophysiology. *Neuroscience and Biobehavioral Reviews*.

[38] Godar S. C., Bortolato M. (2014). Gene-sex interactions in schizophrenia: focus on dopamine neurotransmission. *Frontiers in Behavioral Neuroscience*.

[39] Lindová J., Novotná M., Havlíček J. (2006). Gender differences in behavioural changes induced by latent toxoplasmosis. *International Journal for Parasitology*.

[40] Flegr J., Lindová J., Kodym P. (2008). Sex-dependent toxoplasmosis-associated differences in testosterone concentration in humans. *Parasitology*.

[41] Lue L. F., Rydel R., Brigham E. F. (2001). Inflammatory repertoire of Alzheimer’s disease and nondemented elderly microglia in vitro. *Glia*.

[42] Reveille J. (2004). Predictive value of autoantibodies for activity of systemic lupus erythematosus. *Lupus*.

[43] Dalmau J., Gleichman A. J., Hughes E. G. (2008). Anti-NMDA-receptor encephalitis: case series and analysis of the effects of antibodies. *The Lancet Neurology*.

[44] Monji A., Kato T. A., Mizoguchi Y. (2013). Neuroinflammation in schizophrenia especially focused on the role of microglia. *Progress in Neuro-Psychopharmacology and Biological Psychiatry*.

[45] Pawson C. T., Scott J. D. (2010). Signal integration through blending, bolstering and bifurcating of intracellular information. *Nature Structural and Molecular Biology*.

[46] Brown A. S., Begg M. D., Gravenstein S. (2004). Serologic evidence of prenatal influenza in the etiology of schizophrenia. *Archives of General Psychiatry*.

[47] Gotlieb-Stematsky T., Zonis J., Arlazoroff A., Mozes T., Sigal M., Szekely A. (1981). Antibodies to Epstein-Barr virus, herpes simplex type 1, cytomegalovirus and measles virus in psychiatric patients. *Archives of Virology*.

[48] Dubey J. (2001). Parasitemia and early tissue localization of Sarcocystis neurona in interferon gamma gene knockout mice fed sporocysts. *Journal of Parasitology*.

[49] Lauw F. N., Caffrey D. R., Golenbock D. T. (2005). Of mice and man: TLR11 (finally) finds profilin. *Trends in Immunology*.

[50] Zhang D., Zhang G., Hayden M. S. (2004). A toll-like receptor that prevents infection by uropathogenic bacteria. *Science*.

[51] Kannan G., Prandovszky E., Steinfeldt C. B. (2014). One minute ultraviolet exposure inhibits *Toxoplasma gondii* tachyzoite replication and cyst conversion without diminishing host humoral-mediated immune response. *Experimental Parasitology*.

